# Microporous organic hydroxyl-functionalized polybenzotriazole for encouraging CO_2_ capture and separation†

**DOI:** 10.1039/c9ra03741a

**Published:** 2019-07-22

**Authors:** Qiang Yin, Chunlin Lu, Shuai Zhang, Meifang Liu, Kai Du, Lin Zhang, Guanjun Chang

**Affiliations:** Research Center of Laser Fusion, China Academy of Engineering Physics Mianyang 621900 P. R. China liumeifang@caep.cn; State Key Laboratory of Environment-friendly Energy Materials, School of Material Science and Engineering, Southwest University of Science and Technology Mianyang 621010 P. R. China gjchang@mail.ustc.edu.cn

## Abstract

We report a mild, hydroxyl functionalized and thermal stable benzotriazole-based aerogel (HO-PBTA), which is inspired by phenolic resin chemistry. Taking advantage of the synergistic adsorption interactions between hydroxy-benzotriazole and CO_2_, and the phobic effect between benzotriazole and nitrogen (N_2_), the CO_2_ uptake capacity of the HO-PBTA reaches an encouraging level (6.41 mmol g^−1^ at 1.0 bar and 273 K) with high selectivity (CO_2_/N_2_ = 76 at 273 K).

## Abstract 2

We report a mild, hydroxyl functionalized and thermal stable benzotriazole-based aerogel (HO-PBTA), which is inspired by phenolic resin chemistry.

## Graphical abstract

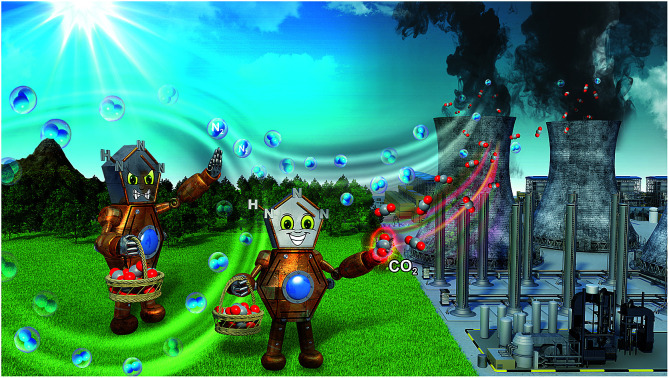

Nowadays, global warming caused by increased concentrations of carbon dioxide (CO_2_) in the atmosphere is one of the most serious environmental problems.^1–3^ The development of novel functional materials and new technologies for CO_2_ capture and storage has gained great attention. Microporous organic polymers (MOPs) with intrinsic properties including large specific surface area, narrow pore size distribution, good chemical stability, and low skeleton density have exhibited potential applications in gas storage and separation.^4–7^ In addition, microporous porous materials with excellent intrinsic properties also made significant breakthroughs in liquid separation.^8,9^ The structure and CO_2_ adsorption of the MOPs have complicated relationships. Therefore, the design of high performance CO_2_ capture materials often involves sophisticated molecular design and careful adjustments of the ingredient ratio. It has been shown that the incorporation of N-containing groups into the pore wall of MOPs has a profound impact on both CO_2_ uptake and selectivity by enhancing their physisorption interactions,^10–15^ however, it still remains a great challenge to make a facile synthesis of functional MOP materials that capture CO_2_ efficiently and selectively.

In the application of CO_2_ capture, the nitrogen-containing MOPs act as capable storage media due to physisorption that involves an electron donor–acceptor mechanism between a heteroatom nitrogen and CO_2_ on the inner surface of the networks.^16–18^ The first principles study indicated that nitrogen-containing heteroaromatic groups can form strong physical interactions with CO_2_*via* “dispersive π–π stacking” and electrostatic “in-plane” mechanisms.^19^ This theoretical calculation guides us how to design the functional materials that capture CO_2_ efficiently and selectively. In previous work, we described a new strategy for CO_2_ capture based on the synergistic effect of electrostatic in-plane and dispersive π–π stacking interactions of two functional groups with CO_2_, and the proposed synergistic effect can be considered as a new rationale for the design and fabrication of CO_2_ capture materials.^20^ In addition, it has been demonstrated that azo-functionalized MOPs exhibit the N_2_ phobicity due to the entropic loss of N_2_ gas molecules upon binding, which endows the networks with the unprecedented CO_2_ selectivity.^21^ Inspired by these reported studies, we hypothesized that both the CO_2_ adsorption capacity and CO_2_/N_2_ selectivity can be improved greatly by involving multiple, more than two, functional groups in MOP networks where multiple mechanisms work for CO_2_ capture and separation. In this work, the synergistic adsorption interactions between the HO-PBTA polymer and CO_2_ molecules, the N_2_-phobic effect between the HO-PBTA polymer and N_2_ have been investigated in details. It is expected that the comprehensive effects of the CO_2_-philic and N_2_-phobic behaviors will provide new design principles for the development of next-generation functional porous polymers with high CO_2_ adsorption capacity and selectivity.

To achieve this objective, a benzotriazole-based microporous aerogel (HO-PBTA), with azo, hydroxyl and imino groups in the polymer chains, was fabricated *via* sol–gel technology involving phenolic resin-inspired chemistry^22–24^ and followed by CO_2_ supercritical drying (Fig. 1A). The material preparation and characterization are detailed in the ESI.† The as-prepared HO-PBTA is a dark gray, porous ultralight material, as shown in Fig. 1B. HO-PBTA aerogel was characterized by Fourier transform infrared and ^13^C CP/MAS NMR, and the results were in good agreement with the proposed structures (Fig. 2). The FTIR spectrum of the HO-PBTA is shown in Fig. 2a, in which the absorption peaks at about 3190 cm^−1^ and 3402 cm^−1^ correspond to the structure of NH and the OH groups. The peak at 1618 cm^−1^ is attributed to the vibration of the aromatic ring skeleton. And the absorption at 1536 cm^−1^ corresponds to the structure of C=N in the network. As shown in Fig. 2B, the broad peaks at 150–110 ppm are ascribed to the benzotriazole group carbons, and the peaks at 75–25 ppm corresponds to methylene carbons.

**Fig. 1:**
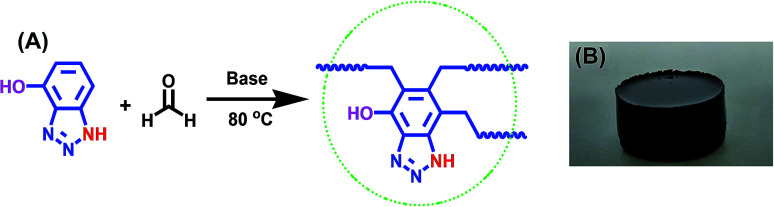
Synthesis of HO-PBTA aerogel. (A) Dark gray aerogel of HO-PBTA was obtained by reacting 4-hydroxy-1*H*-benzotriazole with formaldehyde, in water at 80 °C in the presence of potassium hydroxide under air conditions; (B) the photograph of the HO-PBTA aerogel.

**Fig. 2:**
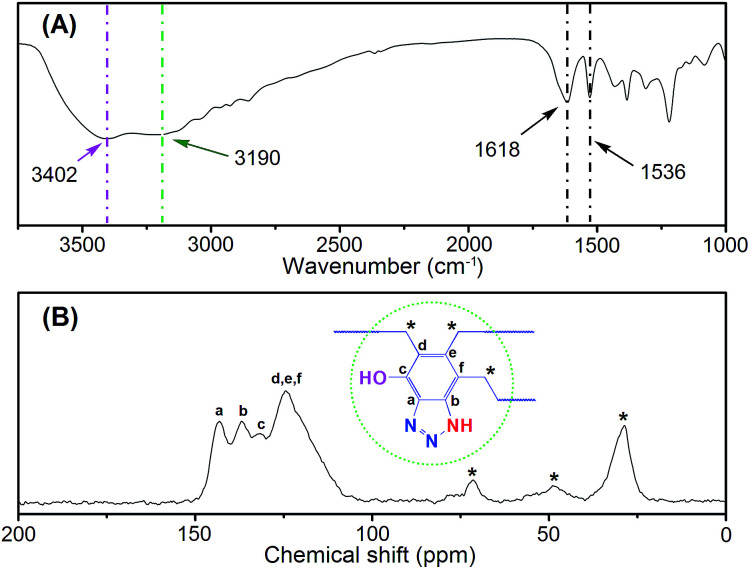
Chemical structure characterization of the HO-PBTA polymer material. (A) FTIR spectrum, recorded as KBr pellet; (B) ^13^C CP/MAS NMR spectrum.

The porosity of the HO-PBTA aerogel was quantified by scanning electron microscopy (SEM), transmission electron microscopy (TEM), and sorption analysis. A SEM image shows that the HO-PBTA aerogel consists of aggregated particles with submicrometer sizes (Fig. 3A). The TEM image (Fig. 3B) reveals the micropore structure, which is an essential requirement for CO_2_ capture. The porosity of aerogel was further quantified by sorption analysis using nitrogen (N_2_) as the sorbate molecule, and the HO-PBTA aerogel is microporous and exhibits a combination of type I and II N_2_ sorption isotherms according to the IUPAC classification (Fig. 4A).^25^ The increase in the nitrogen sorption at a high relative pressure above 0.9 may arise in part from interparticulate porosity associated with the meso- and macrostructures of the samples and interparticular void.^26^ According to ref. 27, the specific surface areas calculated in the relative pressure (*P*/*P*_0_) range from 0.01 to 0.1 shows that the Brunauer–Emmett–Teller (BET) specific surface area of HO-PBTA is up to 2160 m^2^ g^−1^ (Fig. S3†). The pore size distribution (PSD) of the network calculated from the adsorption branch of the isotherms with the nonlocal density functional theory (NLDFT) approach indicates that HO-PBTA aerogel exhibits a dominant pore diameter centered at about 0.60 nm (inset in Fig. 4A). Thermal property of HO-PBTA aerogel was evaluated *via* thermogravimetric analysis (TGA) in nitrogen and air conditions, and the typical TGA curves are shown in Fig. S4.† HO-PBTA exhibits great thermal stability with high decomposition temperature (*T*_d_, 5% = ∼500 °C) at N_2_ atmosphere. The TGA curve indicated that the HO-PBTA aerogel still exhibited good thermal stability at air condition.

**Fig. 3:**
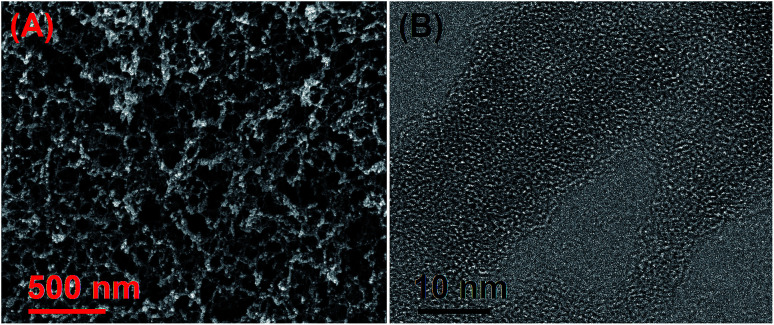
Microstructural characterization of HO-PBTA aerogel. (A) Scanning electron microscopy (SEM) image of HO-PBTA aerogel; (B) transmission electron microscopy (TEM) image of HO-PBTA aerogel.

**Fig. 4:**
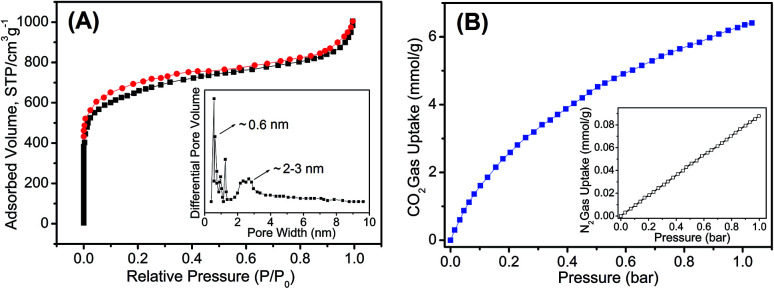
(A) Nitrogen adsorption–desorption isotherms and the pore size distribution calculated by the nonlocal density functional theory (inset) of HO-PBTA aerogel. (B) Gas adsorption isotherms of HO-PBTA aerogel at 273 K.

The CO_2_ adsorption capacity and selectivity (CO_2_/N_2_) in HO-PBTA aerogel were evaluated by adsorption isotherm measurements. As shown in Fig. 4B, the CO_2_ capture exhibits an increase with the increasing of the pressure. The CO_2_ adsorption capacity of the HO-PBTA aerogel is as high as 6.41 mmol g^−1^ at 1.0 bar and 273 K, while the adsorption capacity of N_2_ is only 0.09 mmol g^−1^ at the same conditions (inset in Fig. 4B). The CO_2_ adsorption capacity of the HO-PBTA aerogel is up to 2.3 mmol g^−1^ at 1 bar and 323 K, and 0.02 mmol g^−1^ for N_2_ at the same conditions (Fig. S5†). Equilibrium CO_2_ adsorption capacity is found to decrease with an increase in temperature due to the exothermic nature of the adsorption process, as expected for physical adsorbents. We found that the CO_2_ adsorption capacity of the HO-PBTA aerogel is higher than most of the CO_2_ capture MOP materials, and close to some of the MOF materials (Table S1†).^28–30^ This high affinity is a consequence of the favourable interactions of the polarizable CO_2_ molecules through multiple adsorption interactions with the framework, and also the inherent microporosity of HO-PBTA aerogel. Additionally, HO-PBTA aerogel also has great CO_2_ adsorption capacities and selectivities at 298 and 323 K (Fig. S5†), and these values are still comparable to the high surface area MOP networks.^31–33^

For the further CO_2_ adsorption, CO_2_ was also adsorbed preferentially over N_2_ at high temperatures. The selectivities were calculated using the Ideal Adsorbed Solution Theory (IAST) for CO_2_/N_2_ mixture in the ratio of 0.15 : 0.85. At 1 bar, the IAST CO_2_/N_2_ selectivities of the HO-PBTA were 76 at 273 K, 97 at 298 K and become 110 at 323 K, which close to the highest one reported to date under the same conditions. Additionally, the HO-PBTA aerogel have the high selectivity of CO_2_/N_2_ at high temperature than other porous materials (Table S2†). It is worth noting that the CO_2_/N_2_ selectivities increased with the temperature increasing, which are essential requirements for high temperature post-combustion CO_2_ adsorption. N_2_ uptake drops ∼70% at a temperature increase from 273 to 298 K, compared with a ∼60% drop for CO_2_. This phenomenon is in line with the conventional CO_2_ affinity and the concept of N_2_ phobic in nitrogen-rich porous polymers where nitrogen-rich groups (–N=N–) will reject N_2_ gas selectively.^21^ To further akin to capture from post-combustion gas streams, CO_2_ adsorption capacity of HO-PBTA aerogel at higher temperature have been characterized (Fig. S6†). The HO-PBTA shown the good CO_2_ adsorption capacity of 1.8 mmol g^−1^ at 333 K and 1.5 mmol g^−1^ 343 K. The N_2_ uptakes of HO-PBTA aerogel under the same conditions were 0.016 mmol g^−1^ and 0.015 mmol g^−1^, resulting in selectivity of 112 and 101, respectively.

The isosteric heat of adsorption (*Q*_st_) for HO-PBTA aerogel was calculated using the virial equations.^34^ As shown in Fig. S7,† HO-PBTA has a *Q*_st_ value of 33.9 kJ mol^−1^, and this value can be considered as the optimum for gas adsorption and separation because of a balance between the reversibility and selectivity. The *Q*_st_ values is highest among reported values for organic porous polymers and comparable to some MOFs compounds.^21,35,36^ The impressive *Q*_st_ of 33.9 kJ mol^−1^ further indicates the strong interaction of HO-PBTA polymers with CO_2_ guest molecules. It should be noted that the higher and more optimized *Q*_st_ value of the HO-PBTA aerogel can be ascribed to the synergistic effect of azo (–N=N–), hydroxyl (–OH) and imino (–NH) units arising from different interaction mechanisms.

To illustrate the synergistic adsorption mechanism, we used density functional theory (DFT)^19,20,37–39^ to investigate the interaction of HO-PBTA with CO_2_ and to track the CO_2_ capture process. The calculation is detailed in the ESI. Fig. 5 shows a series of snapshots for CO_2_ capture by 4-hydroxybenzotriazole, as the model compound, where benzotriazole and hydroxyl work synergistically to adsorb multiple CO_2_ molecules. The minimum energy structure of the CO_2_–azo complex is obtained when CO_2_ lies on the azo at a bond distance of 3.06 Å to form the π–π stacking conformation (Fig. 5B). The electrostatic “in-plane” equilibrium conformation of CO_2_–HO-PBTA involves two sites: one is the electron deficient central carbon atom of CO_2_ to the lone pair of electrons on a nitrogen atom of azo group *via* dipole–quadrupole interaction; the other is lone pairs of oxygen on CO_2_ to a hydrogen atom on the imino group (or a hydrogen atom on the hydroxyl) *via* hydrogen bonding (Fig. 5C and F). Either dipole–quadrupole interaction or hydrogen bonding cannot stabilize the CO_2_–HO-PBTA complex because of their low binding energies.^19^ However, previous works and our calculation indicated that simultaneous formation of dipole–quadrupole interaction and hydrogen bonding at both sites cause a much more stable in-plane conformation of CO_2_–HO-PBTA complex (Fig. S8†).^19,20^ However, the capture of flowing CO_2_ by electrostatic “in-plane” interactions is difficult due to a small binding area by only two atomistic sites. On the other hand, CO_2_ can be rapidly adsorbed on the azo group because of its relatively large binding area (Fig. 5A). To our knowledge, the desorption occurs frequently driven by thermal fluctuation. Once CO_2_ desorption, the starting speed should be much slower than the bulk speed, resulting in a high probability to be captured by an adjacent hydroxyl and imino groups. The in-plane conformation of CO_2_–HO-PBTA complex is, therefore, formed much easily and efficiently with help of the functional azo while retaining the high selectivity of CO_2_ over other gas molecules (Fig. 5H). Final coordinates of DFT geometry optimization was shown in Table S3.†

**Fig. 5:**
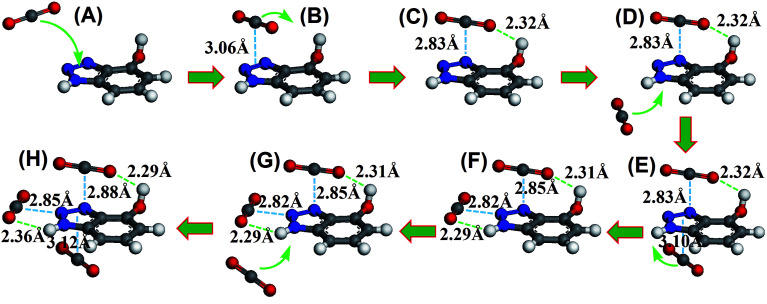
DFT results to track the full CO_2_ capture process. (A) A CO_2_ molecule is adsorbed on the face of an electron-rich azo group *via* the dispersive π–π stacking interaction. (B and C) The desorbed CO_2_ molecule can be captured by an adjacent hydroxyl, a stable “electrostatic in-plane” conformation including dipole–quadrupole and hydrogen bond interactions is formed. (D) The second CO_2_ molecule comes close to the azo group, (E) and the CO_2_ molecule is adsorbed on the azo group. (F) The desorbed CO_2_ molecule can be captured by an adjacent imino group. (G) The third CO_2_ molecule comes close to the azo group, (H) and the CO_2_ molecule is adsorbed on the azo group. The gray, white, blue and red spheres represent C, H, N, and O atoms, respectively.

## Conclusions

In summary, we designed measurements to show that hydroxyl functionalized benzotriazole-based microporous aerogel (HO-PBTA) can efficiently capture CO_2_ with encouraging adsorption capacity and selectivity, which are essential requirements for high temperature CO_2_ adsorption. The key innovation of this work includes three aspects: first, taking advantage of the synergistic adsorption interactions between benzotriazole and CO_2_, and the phobic effect between benzotriazole and nitrogen (N_2_), the CO_2_ uptake capacity of the HO-PBTA reaches an encouraging level (6.41 mmol g^−1^) at 1.0 bar and 273 K, with a high selectivity CO_2_/N_2_ = 76 at 273 K and 110 at high temperature 323 K. Second, in comparison to the reported porous organic polymers, the facile synthetic strategy exhibits cost-effective advantages, making the HO-PBTA network the one of most promising microporous materials for application in CO_2_ capture and separation.

## Conflicts of interest

There are no conflicts to declare.

## Supplementary Material

RA-009-C9RA03741A-s001
