# Supplementary material for: Microporous organic hydroxyl-functionalized polybenzotriazole for encouraging CO2 capture and separation
Source: RSC Adv. 2019 Jul 22;9(39):22604–8. doi: 10.1039/c9ra03741a (PMC9067136; doi:10.1039/c9ra03741a)
Supplement: RA-009-C9RA03741A-s001 [file RA-009-C9RA03741A-s001.pdf]

Electronic Supplementary Material (ESI) for RSC Advances.
This journal is © The Royal Society of Chemistry 2019

Supporting Information

Microporous organic hydroxyl-functionalized polybenzotriazole gel for encouraging CO_2 capture and separation

Qiang Yin,[a] Chunglin Lu,[a] Shuai Zhang,[a] Meifang Liu,*[a] Kai Du,[a] Lin Zhang[a] and Guanjun Chang*[b]

[a] Research Center of Laser Fusion, China Academy of Engineering Physics, 621900, Mianyang, P. R. China.

[b] State Key Laboratory of Environment-friendly Energy Materials & School of Material Science and Engineering, Southwest University of Science and Technology, Mianyang, 621010, P. R. China. gjchang@mail.ustc.edu.cn

*To whom correspondence should be addressed:
Meifang Liu, Email：liumeifang@caep.cn;
GuanjunChang, Email: gjchang@mail.ustc.edu.cn.

The main materials

Benzotriazole (99%) and $HgSO_4$ (98%) was purchased from J & K Technology Co., Ltd., and oleum (wt% of SO_3 = 20%), KOH (99%) and hydrochloric acid (wt% = 37%) were obtained from different commercial sources and used without further purification.

The main measurements

FT-IR spectrum was recorded on a Nicolet 6700 FTIR spectrometer. ^1H NMR and ^{13}C NMR were performed on a Bruker 300MHz NMR spectrometer with dimethylsulfoxide (DMSO-d_6) as the solvent. Solid-state cross-polarization magic-angle-spinning (CP/MAS) NMR spectra were recorded on a Bruker Avance III 400 NMR spectrometer. Thermogravimetric analysis (TGA) was performed on a Setarma TG-92 at a heating rate of 10 °C/min under nitrogen atmosphere. Scanning electron microscopy (SEM) was recorded on an S-4800 (Hitachi Ltd) field emission scanning electron microscope. Morphological observation was performed with a Tecnai G2 F20 S-TWIN (FEI Company) transmission electron microscope (TEM). Gas adsorption isotherms were measured by a volumetric method using a Micromeritics AR-JW-BK112 instrument. The samples were degassed 10 hours at 120 °C, and the obtained adsorption-desorption isotherms were evaluated to obtain the pore parameters, including Brunauer-Emmett-Teller (BET) specific surface area, pore size, and pore volume. The pore size distribution (PSD) was calculated from the adsorption branch with the nonlocal density functional theory (NLDFT) approach. The selectivity of the aerogels to separate CO_2 from CO_2/N_2 mixtures was estimated by the ratio between the CO_2 and N_2 adsorption capacities at a selected pressure. The Clausius-Clapeyron equation was employed to calculate the enthalpies of adsorption for CO_2 on the networks. In each case, the data were fit using the

equation: $(\ln P)_n = -(Q_{st}/R)(1/T) + C$, where P is the pressure, n is thxe amount adsorbed, T is the temperature, R is the universal gas constant and C is a constant. The isosteric heat of adsorption Q_{st} was subsequently obtained from the slope of plots of $(\ln P)_n$ as a function of $1/T$.

Synthesis of 4-SO_3K-BTA: To a one-necked flask equipped with magnetic stirrer, mercury sulfate (0.14 g), oleum (4.6 ml) were added and stirred at room temperature. Afterwards, benzotriazole (5.95 g) was dissolved in oncentrated sulphuric acid (7 ml) and added to one-necked flask and under below 80 °C. The reaction mixture was heated to 130 °C under stirring for 2 h. The resulting solution was allowed to slowly cool to room temperature, and subsequently dropped into 35ml 20 mol/L KOH solution to PH≥7. Afterwards, the white solid precipitated, filtered, washed with water and dried 80 °C under vacuum (13.5 g, 98%).

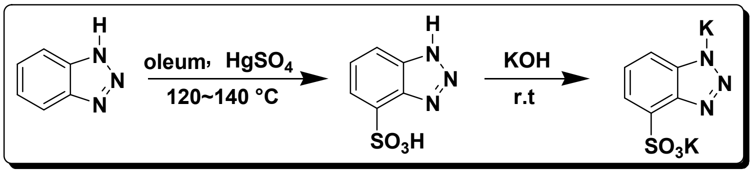

Synthesis of 4-OH-BTA: The autoclave was charged 4-SO_3K-BTA (8.39 g), KOH (6.35 g), H_2O(50 ml), and reaction mixture was heated to 235 °C at high-purity argon and P=4 atm under stirring for 1 h. The reaction solution was cooled to room temperature, and subsequently poured into water, filtered. Afterwards, concentrated HCl was added to the filtrate to adjust the solution pH=1, and obtained light yellow floccule, filtered, washed with water and dried 80 °C under vacuum. The crude material was dissolved in water and mixture was heated to 70 °C until the crude product is completely dissolved, filtered hot

solution. Then solution promptly cool down to room temperature and obtained light yellow product, filtered. Afterwards, the product dried 80 °C under vacuum and obtained light yellow powder (2.5 g, 61%).

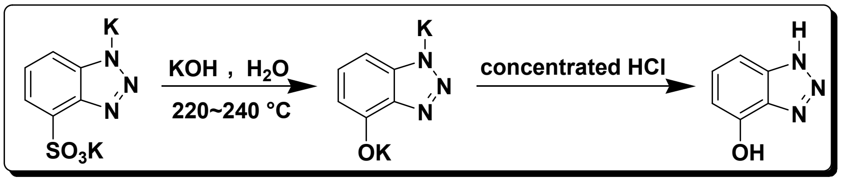

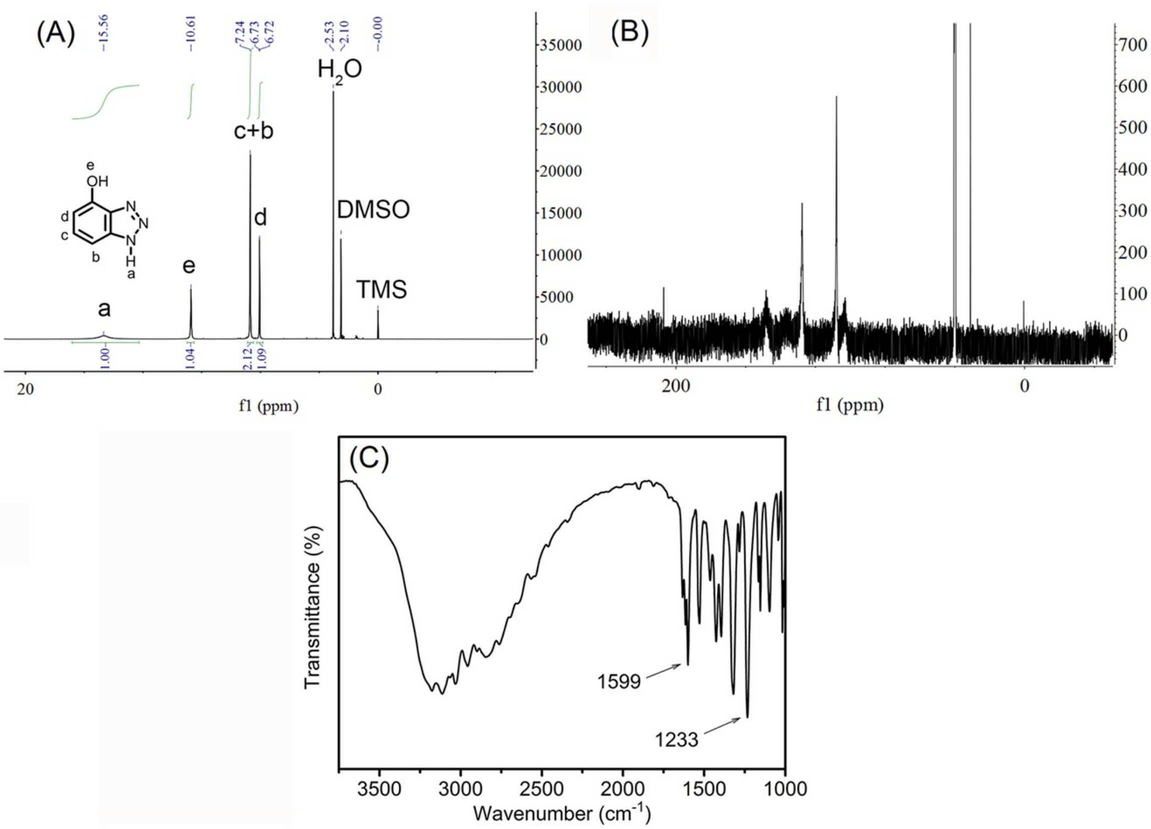

Figure S1. Characterizations of the 4-Hydroxy-1H-benzotriazole (4-OH-BTA). (A) ^{1}H NMR, (B) ^{13}C NMR, (C) FTIR.

Preparation of benzotriazole-based microporous aerogel (HO-PBTA): HO-PBTA aerogel was synthesized by the typical process: 4-OHBTA (0.1351 g, 1 mmol) and KOH (0.0135

g, 0.24 mmol) were dissolved in 2.2 mL deionized water. The reaction mixture were mixed at 80 °C under vigorously stirred at 800 rap for 30 min. Then, HCHO aqueous (150 μL, 37%) was dropwise added to the mixture and formed gel. The resulting gel was aged at 45 °C for 48 h and subsequently soaked with acetone for 3 days to exchange water. The final dried gel was obtained by supercritical dried for 7 days for testing.

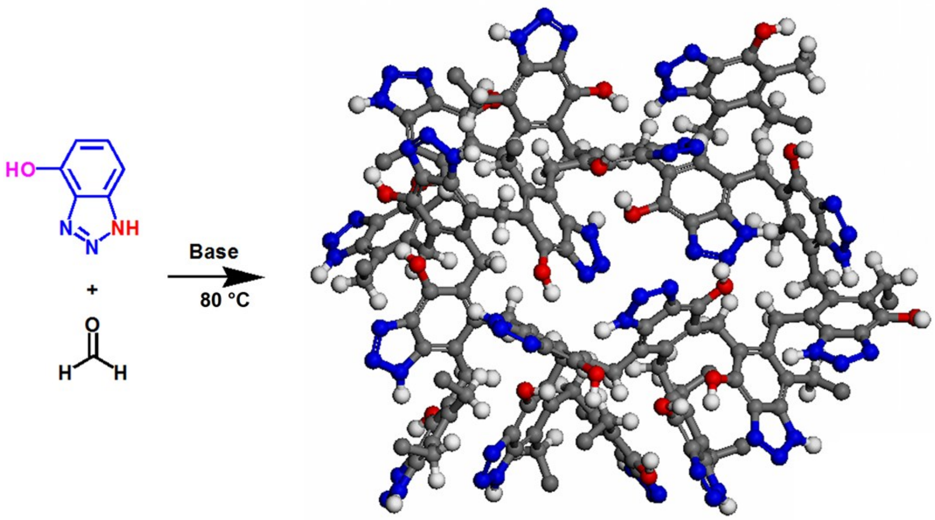

Figure S2. Synthetic route of benzotriazole-based microporous aerogel (HO-PBTA)

BET specific surface area plots of HO-PBTA

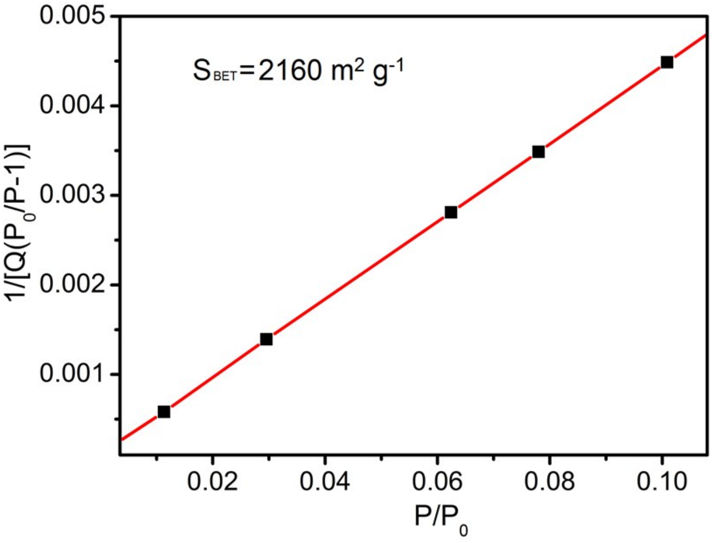

Figure S3. BET specific surface area plots of HO-PBTA aerogel.

Thermal stability of microporous HO-PBTA

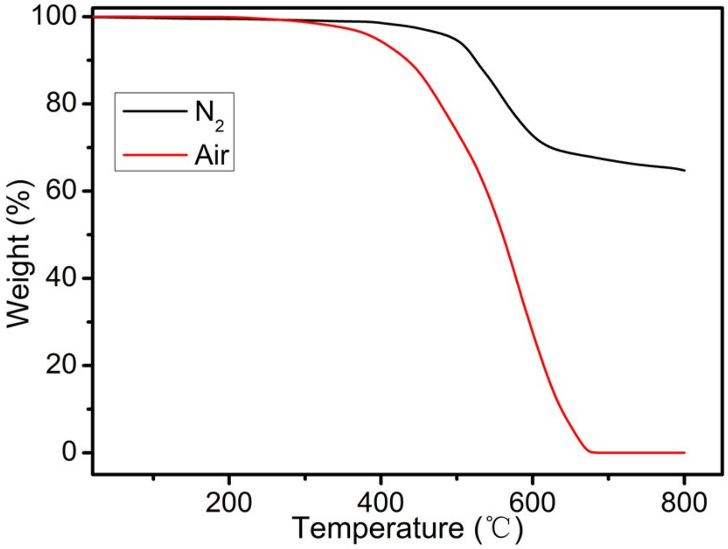

Figure S4. Thermogravimetric analysis (TGA) of HO-PBTA aerogel under nitrogen and air atmosphere up to 800 ^{o}C at a rate of 10 $^{o}C/min$.

Gas adsorption isotherms of HO-PBTA

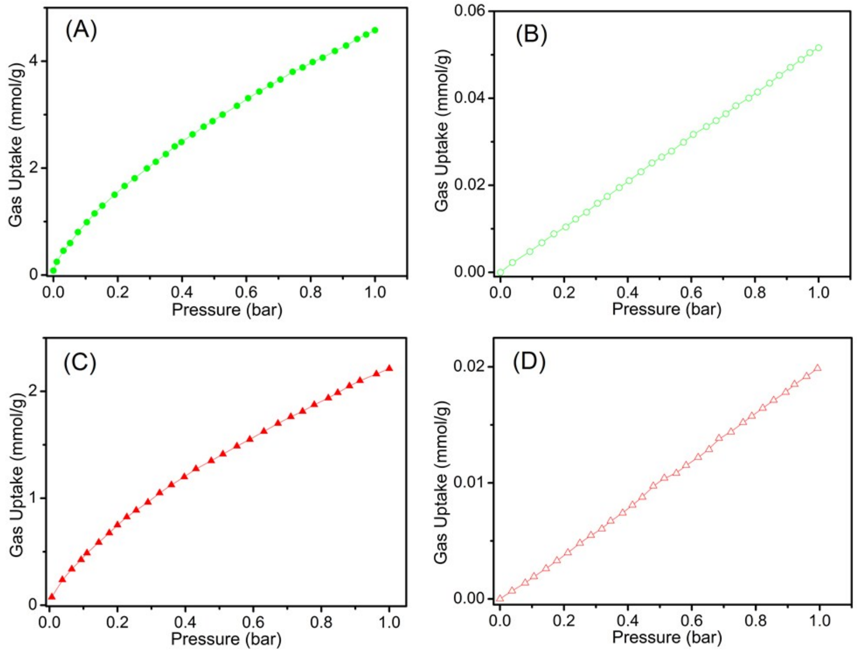

Figure S5. Gas adsorption isotherms of HO-PBTA aerogel. (A) CO_2 at 298 K; (B) N_2 at 298 K; (C) CO_2 at 323K; (D) N_2 at 323K.

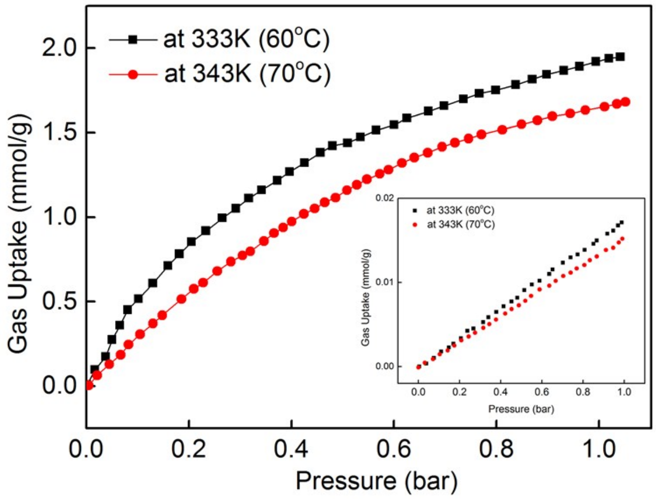

Figure S6. Gas adsorption isotherms of HO-PBTA aerogel at higher temperature (333K; 343K).

Isosteric heat of CO_2 adsorption for HO-PBTA

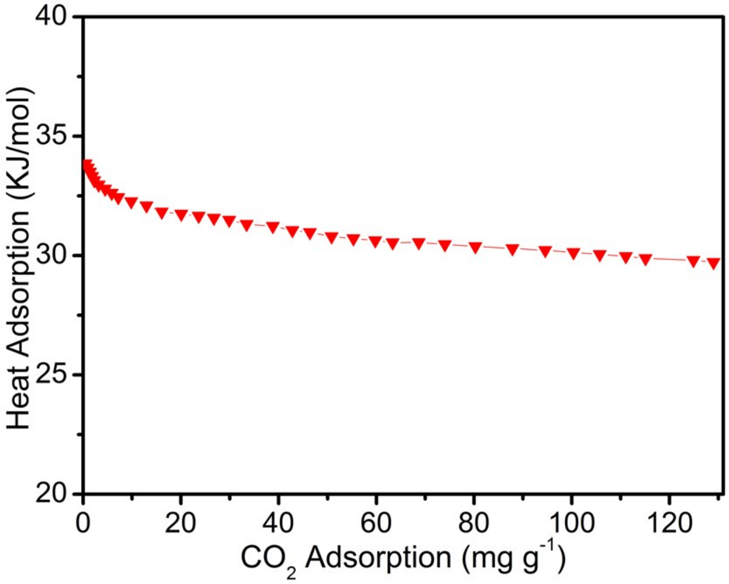

Figure S7. The isosteric heat of CO_2 adsorption for HO-PBTA

Fully optimized geometries of model compound-CO_2 interactions

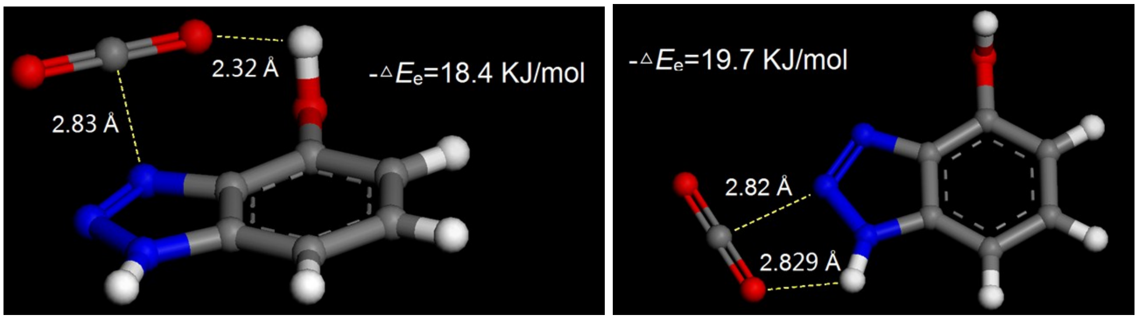

Figure S8. Fully optimized geometries of model compound-CO_2 interactions calculated using density functional theory (DFT).

Table S1. Comparison with state-of-the-art CO_2 capture materials (MOPs and MOFs) in terms of their carbon dioxide capture at 273K.

Related porous materials	CO_2 capture (mmol g^{-1})	Refences
HO-PBTA	6.4	This work
TTMP	3.7	12
N-rich polyme	2.2	13
PMOP	5.0	14
PCN-250 (Fe_2Co)	3.9	15
PCTP-1	4.9	16
PCTP-2	3.5	
PCN-TPC	3.7	17
PCN-TPPC	3.2	
PAN-FMP	3.3	18

PI-opt-C	3.4	19
PBO-M	2.6	20

Table S2. Comparison with other porous materials in terms of their carbon dioxide capture and IAST selectivities at 298K.

Related porous materials	CO_2 capture (mmol g^{-1})		IAST Selectivity	Refences
	273K	298K	CO_2/N_2 (298K)	
HO-PBTA	6.4	4.3	76	This work
ALP-1	5.4	3.3	28	21
ALP-2	4.8	2.5	26	
SU-MAC-500	6	4.5	39	22
COP-19	-	1.8	7.8	23
COP-20	-	1.5	4.5	
FCTF-1	4.67	0.92	31	24
FCTF-1-600	5.53	0.68	19	
TSP-2	4.1	2.6	25	25

Simulation methods

To illustrate the molecular mechanism, we used density functional theory (DFT)[1,2] to investigate the interaction of indole, amide with CO_2 and to track the CO_2 capture process. They were calculated at the M06-2X level with the aug-cc-pVDZ basis set and the resolution-of-identity spin-component-scaling Möller-Plesset second-order perturbation theory (RI-scs-MP2) level with the aug-cc-pVTZ basis set.[3-5] The geometries were fully optimized without symmetry constraints at each calculation level. The M06-2X

functional (hybrid-meta GGA with dispersion correction) has shown good performance in the investigation of the dispersion interaction as well as the electrostatic interaction (H-bonding, H-π interaction, π-π interaction, additional electrostatic and induction energies of neutral and charged dimeric systems).[6,7] Single point calculations using the RI-coupled cluster theory with single, double and perturbative triple excitations (RI-CCSD(T)) were performed by employing the aVTZ and aug-cc-pVQZ (aVQZ) basis sets at the RI-scs-MP2/aVTZ geometries. The CO_2-BEs were calculated at the complete basis set (CBS) limit at the RI-CCSD(T) level with the aVTZ and aVQZ basis sets by employing the extrapolation approximation.[8,9] The complete basis set energies were estimated with the extrapolation scheme utilizing the electron correlation error proportional to N^{-3} for the aug-cc-pVNZ basis set (N=3:T, N=4:Q). It is generally known that the zero-point-energy (ZPE)-uncorrected BE($-\Delta E_e$) is closer to the experimental CO_2-adsorption enthalpy (ΔH_{ads}) than the ZPE-corrected BE($-\Delta E_0$).[10,11] Therefore, the values of $-\Delta E_e$ are reported as the CO_2-BEs.

Table S3. Final coordinates (Angstroms) of DFT geometry of Fig. 5H.

	ATOM	X	Y	Z
1	C	-3.932934	-2.290193	0.039896
2	C	-3.101824	-3.420899	0.097029
3	C	-1.726019	-3.302504	0.093848
4	C	-1.202338	-2.003034	0.021523
5	C	-2.048436	-0.886853	-0.023988
6	C	-3.439606	-0.996525	-0.017107
7	N	-1.160499	0.150107	-0.062213
8	N	0.1188	-0.297827	-0.029046
9	N	0.104204	-1.585217	0.016489
10	H	-5.002506	-2.443847	0.046492
11	H	-3.533199	-4.40916	0.144164
12	H	-4.090647	-0.137973	-0.060809

13	H	-1.308366	1.144783	-0.092946
14	O	-0.934541	-4.397453	0.162024
15	H	-0.012244	-4.119018	0.236036
16	O	1.689106	-1.144433	3.195431
17	C	1.35445	-2.2239	2.918218
18	O	1.019316	-3.303368	2.644786
19	O	2.847925	1.704591	-0.115508
20	C	1.787832	2.183012	-0.100541
21	O	0.741104	2.69911	-0.083992
22	O	-0.380355	0.143152	-3.503218
23	C	-0.768743	-0.954405	-3.567137
24	O	-1.163494	-2.048258	-3.637516

Reference

[1] Altarawneh, S.; Behera, S.; Jena, P.; El-Kaderi, H. M. New Insights into Carbon Dioxide Interactions with Benzimidazole-Linked Polymers. *Chem. Commun.* **2014**, *50*, 3571.

[2] Mercado, R.; Vlaisavljevich, B.; Lin, L.; Lee, K.; Lee, Y.; Mason, J. A.; Xiao, D. J.; Gonzalez, M. I.; Kapelewski, M. T.; Neaton J. B.; Smit, B. Force Field Development from Periodic Density Functional Theory Calculations for Gas Separation Applications Using Metal-Organic Frameworks. *J. Phys. Chem. C* **2016**, *120*, 12590.

[3] Gerenkamp, M.; Grimme, S. Spin-Component Scaled Second-Order Møller-Plesset Perturbation Theory for the Calculation of Molecular Geometries and Harmonic Vibrational Frequencies. Chem. *Phys. Lett.*, **2004**, *392*, 229.

[4] Zhao, Y.; Truhlar, D. G. Comparative DFT Study of van der Waals Complexes: Rare-Gas Dimers, Alkaline-Earth Dimers, Zinc Dimer, and Zinc-Rare-Gas Dimers. *J. Phys. Chem. A*, **2006**, *110*, 5121.

[5] Grimme, S. Improved second-order Møller-Plesset Perturbation Theory by Separate Scaling of Parallel- and Antiparallel-Spin Pair Correlation Energies. *J. Chem. Phys.*, **2003**, *118*, 9095.

[6] Kolaski, M.; Kumar, A.; Singh, N. J.; Kim, K. S. Differences in Structure, Energy,

and Spectrum between Neutral, Protonated, and Deprotonated Phenol Dimers: Comparison of Various Density Functionals with AB Initio Theory. *Phys. Chem. Chem. Phys.*, **2011**, *13*, 991;

[7] Lee, H. M.; Youn, I. S.; Saleh, M.; Lee, J. W.; Kim, K. S. Interactions of CO_2 with Various Functional Molecules. *Phys. Chem. Chem. Phys.*, **2015**, *17*, 10925.

[8] Min, S. K.; Lee, E. C.; Lee, H. M.; Kim, D. Y.; Kim, D.; Kim, K. S. Complete Basis Set Limit of Ab Initio Binding Energies and Geometrical Parameters for Various Typical Types of Complexes. *J. Comput. Chem.*, **2008**, *29*, 1208.

[9] Helgaker, T.; Klopper, W.; Koch, H. Noga, J. Basis-Set Convergence of Correlated Calculations on Water. *J. Chem. Phys.*, **1997**, *106*, 9639.

[10] Saleh, M.; Lee, H. M.; Kemp, K. C.; Kim, K. S. Highly Stable CO_2/N_2 and CO_2/CH_4 Selectivity in Hyper-Cross-Linked Heterocyclic Porous Polymers. *ACS Appl. Mater. Interfaces,* **2014**, *6*, 7325.

[11] Vaidhyanathan, R.; Iremonger, S. S.; Shimizu, G. K. H.; Boyd, P. G.; Alavi, S.; Woo, T. K. Direct Observation and Quantification of CO_2 Binding Within an Amine-Functionalized Nanoporous Solid. *Science*, **2010**, *330*, 650.

[12] Bera, R.; Ansari, M.; Mondal, S.; Das, N. *Eur. Polym. J.*, **2018**, *99*, 259.

[13] Cui, Y.; Du, J.; Liu, Y.; Yu, Y.; Wang, S.; Pang, H.; Liang, Z.; Yu, J. *Polym. Chem.*, **2018**, *9*, 2643.

[14] Luo, S.; Zhang, Q.; Zhang, Y.; Weaver, K. P.; Phillip, W. A.; Guo, R. *ACS Appl. Mater. Interfaces*, **2018**, *10*, 15174.

[15] Bazaka, K.; Jacob, M. V.; Crawford, R. J.; Ivanova, E. P. *Appl. Microbiol. Biot.*, **2012**, *95*, 299.

[16] Puthiaraj, P.; Kim, S.-S.; Ahn, W.-S. *Chem. Eng. J.*, **2016**, *283*, 184.

[17] Deng, G.; Wang, Z. *ACS Appl. Mater. Interfaces*, **2017**, *9*, 41618.

[18] Li, G.; Zhang, B.; Wang, Z. *Macromolecules*, **2016**, *49*, 2575.

[19] Liao, Y.; Weber, J.; Faul, C. F. J. *Macromolecules*, **2015**, *48*, 2064.

[20] Zhang, B.; Yan, J.; Wang, Z.; *J. Phys. Chem. C*, **2018**, *122*, 12831.

[21] Arab, P.; Rabbani, M. G.; Sekizardes, A. K.; İslamoğlu, T. and El-Kaderi, H. M. *Chem. Mater.* **2014**, *26*, 31385-1392.

[22] To, John W. F.; He, J.; Mei, J.; Haghpanah, R.; Chen, Z.; Kurosawa, T.; Chen, S.; Bae, W.-G.; Pan, L.; Tok, J. B.-H.; Wilcox, J. and Bao, Z. *J. Am. Chem. Soc.* **2016**, *1383*, 1001-1009.

[23] Xiang, Z.; Mercado, R.; Huch, J. M.;Wang, H.; Guo, Z.; Wang, W.; Cao, D.; Haranczyk, M. and Smit, B. *J. Am. Chem. Soc.* **2015**, *137*, 13301-13307.

[24] Zhao, Y.; Yao, K. X.; Teng, B.; Zhang, T. and Han, Y. *Energy Environ. Sci.* **2013**, *6*, 3684-3692.

[25] Zhu, X.; Mahurin, S. M.; An, S.-H.; Do-Thanh, C.-L.; Tian, C.; Li, Y.; Gill, L. W.; Hagaman, E. W.; Bian, Z.; Zhou, J.-H.; Liu, H. and Dai, S. *Chem. Commun.* **2014**, *50*, 7933-7936.
